# Protective effect of quercetin on kidney diseases: From chemistry to herbal medicines

**DOI:** 10.3389/fphar.2022.968226

**Published:** 2022-09-02

**Authors:** Yi-Qin Chen, Hao-Yin Chen, Qin-Qi Tang, Yi-Fan Li, Xu-Sheng Liu, Fu-Hua Lu, Yue-Yu Gu

**Affiliations:** ^1^ Department of Nephrology, Guangdong Provincial Hospital of Chinese Medicine, The Second Affiliated Hospital of Guangzhou University of Chinese Medicine, Guangzhou, China; ^2^ Department of Pharmacology, School of Pharmaceutical Sciences, Guangzhou University of Chinese Medicine, Guangzhou, China

**Keywords:** quercetin, natural product, herbal medicine, kidney injury, renal disease

## Abstract

Kidney injuries may trigger renal fibrosis and lead to chronic kidney disease (CKD), but effective therapeutic strategies are still limited. Quercetin is a natural flavonoid widely distributed in herbal medicines. A large number of studies have demonstrated that quercetin may protect kidneys by alleviating renal toxicity, apoptosis, fibrosis and inflammation in a variety of kidney diseases. Therefore, quercetin could be one of the promising drugs in the treatment of renal disorders. In the present study, we review the latest progress and highlight the beneficial role of quercetin in kidney diseases and its underlying mechanisms. The pharmacokinetics and bioavailability of quercetin and its proportion in herbal medicine will also be discussed.

## 1 Introduction

Kidney diseases are one of the life-threatening diseases with high mortality rates (Li et al., 2021b). Renal injuries could be triggered by various insults such as nephrotoxins, oxidative stress, or inflammation. These pathogenic factors act as the major driving force to promote renal injuries towards fibrosis (Gu et al., 2020b), which may eventually lead to chronic kidney disease (CKD) or end-stage renal disease (ESRD). To date, the effective drugs and therapeutic strategies for renal injury are still limited.

Natural products have been used in the clinical management of the renal disease. The constituent compounds of herbal medicine receive considerable attention in experimental models of kidney disease both *in vivo* and *in vitro* (Chen et al., 2018). Quercetin is one of the most abundant flavonoids present in natural plants. Due to its antioxidative, anti-hypertensive, and anti-diabetic effects, quercetin has been suggested as an effective flavonoid that plays a beneficial role in the treatment of cancer, cardiovascular disease, and metabolic disease (Sok Yen et al., 2021).

Although quercetin has been studied in many studies, we could not locate a recent overview of quercetin’s action in kidney diseases. In the present review, we discuss and explore the biological effects of quercetin on kidney injuries such as nephrotoxicity, renal inflammation, fibrosis, hyperglycemia damage, and oxidative stress. We also identified the pathogenic mechanisms of renal disease and focused on the signaling pathways that are potentially associated with quercetin treatment.

## 2 Pharmacokinetics and bioavailability of quercetin

Quercetin, also known as 3,5,7,3′,4′-pentahydroxyflavone, is a natural flavonoid compound. In nature, it exists in various forms in different plants and can be found as either quercetin aglycone or derivatives, while the most abundant form in the diet is glycosides (Owumi et al., 2019). Quercetin is highly soluble in lipids and alcohol. Due to its hydrophobicity, quercetin has relatively poor solubility in water (0.17–7 μg/ml), gastric fluids (5.5 μg/ml) and small intestine fluids (28.9 μg/ml), which have reduced its bio-accessibility (Bağdatlıoğlu, 2016). Quercetin aglycone exhibit a poor oral bioavailability of about 2%. However, depending on different radicals bound to the quercetin aglycone backbone, the solubility and biochemical activity of quercetin derivatives vary. The glycoside is much more soluble compared to aglycone, as the glycosyl group increases the water solubility. After the intake of quercetin-rich supplements in human bodies, quercetin quickly disappeared in the body with a 1–2 h removal half-life (Graefe et al., 1999).

**FIGURE 1:**
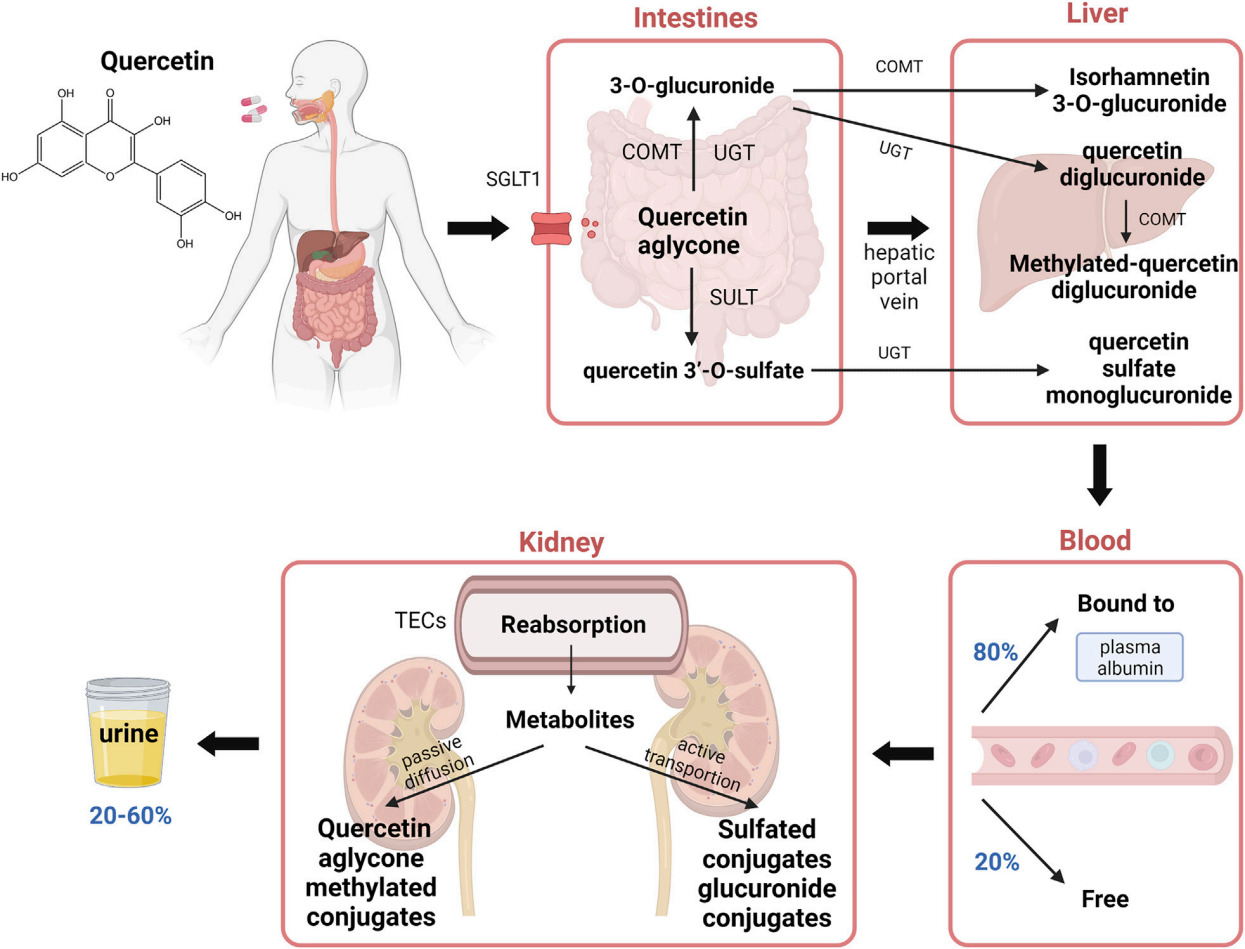
Chemical structure of quercetin and its pharmacokinetics in the body. Quercetin is transported by SGLT1, the process begins with hydrolysis in the gastrointestinal tract and releases quercetin aglycone. Biotransformation reactions include glucuronidation, methylation, and sulfation of quercetin aglycone are catalyzed by UGT, COMT, and SULT, respectively. Main metabolites such as 3-O-glucuronide and quercetin 3′-O-sulfate undergo a second transformation in the liver and 80% of the metabolites are bound to plasma albumin and the remaining 20% are free to enter tissues. The metabolites are reabsorbed in the TECs and enter cells by passive diffusion or active transportation. Up to 20%–60% of the quercetin intake may be secreted into the urine. Abbreviations: SGLT1, sodium-dependent glucose transporter 1; UGT, UDP-glucuronyltransferases; COMT, catechol O-methyltransferases; SULT, sulfotransferases; TECs, tubular epithelial cells.

Quercetin can be transported through sodium-dependent glucose transporter 1(SGLT1). The process begins with the hydrolysis of quercetin glycosides by lactase phloridzin hydrolase (LPH) and intracellular *ß*-Glucosidases and releases quercetin aglycone. Quercetin aglycone is then primarily metabolized in the gastrointestinal tract (Graf et al., 2006). In enterocytes, the biotransformation of quercetin includes glucuronidation by UDP-glucuronyltransferases (UGT), methylation by catechol O-methyltransferases (COMT), and sulfation by sulfotransferases (SULT). 3-O-glucuronide and quercetin 3′-O-sulfate are the two main metabolites passing from the enterocyte and diffusing into the hepatic portal vein to undergo a second transformation in the liver before returning to the bloodstream (Figure 1). Afterward, about 80% of aglycone or metabolites are bound to plasma albumin and the remaining 20% free form can enter the tissues, therefore the aglycone amount is relatively low in the blood. The metabolism process takes place in the intestines, liver and kidneys and the accumulation tends to happen in those organs.

In the kidney, the metabolites from the plasma go through the glomerular filtration process, followed by dispersion into the tubular. The metabolites are partially reabsorbed by tubular epithelial cells (TECs), and the remaining part passes into the urine. The transportation to proximal TECs primarily occurs in the basolateral membrane and apical membrane (Wong et al., 2011). Quercetin aglycone and methylated conjugates across the basolateral membrane by passive diffusion, while sulfated conjugates and glucuronide conjugates use active transport to enter the cells, due to their high affinity for organic anion transporters (OATs). The metabolites are formed in the tubular cells and secreted into the urine. In the human body, the quercetin metabolites excreted through urine take up 20%–60% of total quercetin intake and they are mainly composed of monoglucuronide sulfates, methylated quercetin monoglucuronides, and quercetin diglucuronide (Graf et al., 2006; Mullen et al., 2006).

Previous experiments and studies have revealed the pharmacodynamics of quercetin, it is found that due to its chemical structure, it has low water solubility, oral absorption rate, rapid elimination, and low bioavailability (Diniz et al., 2020). Such characteristics greatly hindered the application of quercetin in pre-employment drug testing and clinical practice (Heeba and Mahmoud, 2016). Casanova et al. (2021) encapsulated quercetin with Pluronic F127 to make micelles and found that it had higher water solubility with good bioavailability, and the protective effect on the kidney had been greatly improved (Gu et al., 2020a). Although more in-depth drug experiments and clinical trials are needed, it is believed that the utilization of quercetin can be improved in the future.

## 3 Quercetin in traditional herbal medicines

Except for various food and supplements, quercetin is widely abundant in flowers, leaves and fruits of plants. It was determined in nearly 200 kinds of traditional Chinese herbal medicines, such as *Sophora japonicum, Radix Bupleuri* (Sen-ming, 2013), *Gynostemmae Pentaphylli Herba* (Conglei Pan, 2019).

High-performance liquid chromatography (HPLC) was mainly used to determine the content of quercetin in herbal medicine. As shown in Table 1, the content of quercetin in different species can vary from less than 1 mg/g to more than 300 mg/g. According to the theory of traditional Chinese medicine (TCM), the efficacy of these herbal medicines containing quercetin can be summarized as follows: 1) heat clearing: the heat described in TCM is somehow related to the inflammatory response in the body and due to quercetin’s significant antioxidant property, it can relieve pain and inflammation. For instance, stranguria is a common urologic disease that is considered to cause by excessive damp heat in the lower energizer. In clinical practice, *Houttuyniae cordata* (Arky Jane Langstieh et al., 2021), *Pyrrosiae lingua* (Chen Junhua et al., 2014)*,* and *Centella asiatica* (Mohammad Azmin and Mat Nor, 2020) are frequently selected, which contained 315.8 mg/g, 234.6 mg/g, and 77.6 mg/g quercetin, respectively. Other representative herbal medicines with heat-clearing effect include *Mori follum*, *Sophora japonica* L., and *Fallopia multiflora Herba* (Vetrova et al., 2017), and *Fallopia multiflora Herba* (Bao Lidao et al., 2015); 2) urination promotion, swelling, or edema reduction: by dilating the renal arteries, quercetin can increase blood and urine volume, therefore alleviating the edema. The common ingredients are *Ephedra Herba* (Saida Ibragic, 2015), *Hedysarum Multijugum Maxim* (Fu Juan and Huang, 2013)*,* and *Plantaginis Semen* (Cao Xuesong and Huang, 2019); 3) promoting kidney recovery: other herbal medicines also exert a nourishing and strengthening effect on the kidney, such as *Lycii Fructus* (Kim Le and Ng, 2007), *Herba Taxilli* (Zhu Kaixin et al., 2011), and *Rubi Fructus* (Zhang Jing and Yan, 2020). *Crataegi Folium* (Deng Ting et al., 2021) (12.73 mg/g) was reported to attain a cardiovascular protection effect by lowering blood lipid, while the contents of quercetin in *Ginkgo Folium* (Qiu et al., 2017) and *Inulae Flos* (Hongmei, 2008) are relatively low (less than 1 mg/g).

**TABLE 1:** The content of quercetin commonly used in traditional Chinese medicine.

Name	Latin name	Active portion	Quercetin content (mg/g)	Reference
Chai Hu	*Radix Bupleuri*	Root	1.7127	Sen-ming (2013)
Che Qian Zi	*Plantaginis Semen*	Seed	0.81	Cao Xuesong and Huang (2019)
Fu Pen Zi	*Rubi Fructus*	Fruit	0.9451	Zhang Jing and Yan (2020)
He Shou Wu	*Fallopia multiflora Harald*	Aerial part	0.55	Bao Lidao et al. (2015)
Huai hua	*S. japonica L.*	Flower	13.7	Vetrova et al. (2017)
Jiao Gu Lan	*Gynostemmae Pentaphylli Herba*	Leaf	14.78	Conglei Pan (2019)
Sang Ji Sheng	*Herba Taxilli*	Leaf	5.27	Zhu Kaixin et al. (2011)
Sang Ye	*Mori Follum*	Leaf	1.784–3.645	Zhong Yuekui and Qiu (2021)
Shan Zha Ye	*Crataegi Folium*	Leaf	12.73	Deng Ting et al. (2021)
Yu Xing Cao	*H. cordata*	Leaf	315.8	Arky Jane Langstieh et al. (2021)
Yin Xing Ye	*Ginkgo Folium*	Leaf	0.609	Qiu et al. (2017)
Xuan Fu Hua	*Inulae Flos*	Flower	0.86	Hongmei (2008)
Gou Qi Zi	*Lycii Fructus*	Fruit	0.296	Kim Le and Ng (2007)
Huang Qi	*Hedysarum Multijugum Maxim*	Root	0.6–1.1	Fu Juan and Huang (2013)
Ji Xue Cao (Asiatic Pennywort Herb)	*C. asiatica (L.)* (Hydro-Cotyle *Asiatica L.*)	Leaf	77.6 (dry)	Mohammad Azmin and Mat Nor (2020)
Ma Huang	*Ephedra Herba*	Stem	2.8 (dry)	Saida Ibragic (2015)
Shi Wei	*P. lingua*	Leaf	234.6	Chen Junhua et al. (2014)

## 4 Renal protective effects of quercetin in kidney disease

### 4.1 Nephrotoxicity

When exposed to certain toxic substances or harmful pollution for a long period, one may occur nephrotoxicity. Due to the special biological structure and physiological role, the kidneys are important organs for drug metabolism and are susceptible to toxins including antineoplastics, antibiotics and many kinds of agents. As summarized in Table 2, many experimental studies and mechanism exploration of multifaceted signal transduction and pathways suggest that quercetin has great potential in reducing renal toxicity.

**TABLE 2:** Protective effects and mechanism of quercetin against renal toxins.

Toxins	Model	Quercetin Dose (mg/kg)	Effects/Mechanisms	References
Cisplatin		50, 100	Anti-inflammatory, maintained renal blood flow, anti-oxidative and enhanced the antitumor activity, reduced renal injury	Sánchez-González et al. (2017), Casanova et al. (2021), Li et al. (2016a), Almaghrabi (2015)
Methotrexate		15, 50	Anti-oxidative, reduced renal injury, scavenged free radicals	Yuksel et al. (2017)
Cyclophosphamide		50	Anti-inflammatory, anti-oxidative	Ebokaiwe et al. (2021)
Doxorubicin		10, 50	Anti-oxidative, anti-inflammatory, protected podocytes	Khalil et al. (2018), Heeba and Mahmoud (2016)
Cadmium		10, 50	Anti-inflammatory, anti-oxidative, reduced renal injury, regulated the metabolism of lipids, amino acids, and purine, anti-oxidative	Jia et al. (2020), Liu et al. (2020), Guan et al. (2021)
Sodium nitrite		200	Anti-inflammatory	Alshanwani et al. (2020)
Diesel exhaust particles		60	Anti-oxidative, anti-inflammatory, promoted autophagy	Morsi et al. (2022)
Ferrous sulfate		50	Reduced renal injury	Gholampour and Saki (2019)
Acrylamide	rats	5, 10, 20, 40, 50	Reduced urea, uric acid levels, anti-oxidative, anti-apoptotic	Bao et al. (2017), Uthra et al. (2017), Bo et al. (2018)
NTiO_2_		75	Anti-inflammatory, anti-oxidative, anti-apoptotic	Alidadi et al. (2018)
Gold nanoparticles		100	Anti-inflammatory, anti-oxidative	Abdelhalim et al. (2018)
Organophosphate pesticides		10, 50	Regulated the metabolism of fatty acids, energy, and sex hormones, anti-oxidative, anti-apoptotic	Qi et al. (2017), Li et al. (2016b)
Ochratoxin A		50	Anti-inflammatory, anti-oxidative, anti-apoptotic	Abdel-Wahhab et al. (2017)
Combination antiretroviral therapy		50	Anti-inflammatory, anti-oxidative, improved the cytoarchitecture and biochemical activities of the organs	Gu et al. (2020a)
Acetaminophen		50	Anti-inflammatory, anti-oxidative, reduced renal injury	Dallak et al. (2020)
Echis pyramidum venom		10	Anti-oxidative, anti-edema, and wound healing effects	Al-Asmari et al. (2018)
Gentamicin		50	Attenuated lipid peroxidation, antioxidative, reduced renal injury	Rahdar et al. (2021)
Valproic acid	Supernatant, renal, tissue	0.05 mM	Cleaned the free radicals, anti-oxidative	Chaudhary et al. (2015)
Contrast media	Human	500 mg	Reduced renal injury	Vicente-Vicente et al. (2019)
	HK-2 cells	10, 100 μm	Reduced renal injury	Andreucci et al. (2018)

Antineoplastic agents such as cisplatin (Li et al., 2016a), methotrexate (Erboga et al., 2015), doxorubicin (Heeba and Mahmoud, 2016), and cyclophosphamide may cause side effects in clinical treatment due to dose-related nephrotoxicity. The nephroprotective effect of quercetin against cisplatin-induced oxidative stress was demonstrated by Almaghrabi (2015). In cisplatin-treated rats, quercetin can reduce tubular injury, downregulate the pro-inflammatory mediators and maintain renal blood flow. Moreover, quercetin also exhibited antioxidant and anti-apoptotic effects, therefore reducing the apoptosis of non-tumor cells caused by cisplatin treatment (Almaghrabi, 2015; Casanova et al., 2021). It is worth mentioning that quercetin did not interfere with the antitumor activity of cisplatin (Sánchez-González et al., 2017). Furthermore, experimental results have shown that quercetin may enhance the activity of cisplatin against cancer (Li et al., 2016a). Likewise, quercetin may protect against cyclophosphamide-induced hepatic and renal injury by immunosuppressing the IDO/TDO pathway (Ebokaiwe et al., 2021). It is hypothesized that this effect may be due to the combination of quercetin’s ability to scavenge reactive oxygen species (ROS) and inhibition of malondialdehyde (MDA) formation. The production of free radicals and ROS are key triggers for the activation of Nrf2 (nuclear factor erythroid 2-related factor 2) and HO-1 (renal heme oxygenase 1). Regarding nephrotoxicity, Nrf2/HO-1 pathway may play an important role in boosting the GSH, GPx, and SOD antioxidant moieties (Arab et al., 2021). Quercetin supplementation could markedly activate the mRNA expression of Nrf2 and HO-1 in copper sulfate-induced renal injury mice (Peng et al., 2020).

Dosage is of great importance in the understanding of the pharmacological effects of quercetin. Of note, evidence also support that when applied with high dose, such as 100 mg/kg/d, quercetin did not show significant improvement in renal function or protection against doxorubicin-induced renal injury (Heeba and Mahmoud, 2016). Nevertheless, quercetin protects kidneys against antineoplastic drugs through the inhibition of inflammatory response, enhancement of the antioxidant system, and exertion of anti-apoptotic effects.

Oral pretreatment of quercetin in rats with gentamicin-induced renal injury (50 mg/kg) for 10 days revealed an improvement in renal injury. The mechanisms of the protective effect of quercetin could be the rebalancing of the antioxidant system and the modulation of renal biomarkers (Rahdar et al., 2021). A study reported by Dallak et al. (2020) showed that toxic doses of acetaminophen formed severe damage to glomerular ultrastructural compartments after 24 h, and apoptosis was observed in renal tissues. Pretreatment with resveratrol and quercetin exerted a protective effect, namely the reduction of p53 expression in the renal tissue, as well as the decrease of blood urea, creatinine, and oxidative biomarkers.

In addition, quercetin also exerts renoprotective effects on antiretroviral combination therapy involving multiple drugs. Valproic acid (VPA) is widely used to intervene in epilepsy and control multiple seizures. It was deduced from experimental studies that the effectiveness of quercetin in protecting against VPA-induced kidney injury and toxicity relies on its ability to scavenge free radicals and alter antioxidant status (Chaudhary et al., 2015). Quercetin showed the potential to improve kidney damage caused by cArt through inhibiting oxidative stress and inflammatory processes. As a result, quercetin participates in the scavenging of toxins, improves the cellular structure of organs and maintains normal biological chemical activity (Gu et al., 2020a).

**FIGURE 2:**
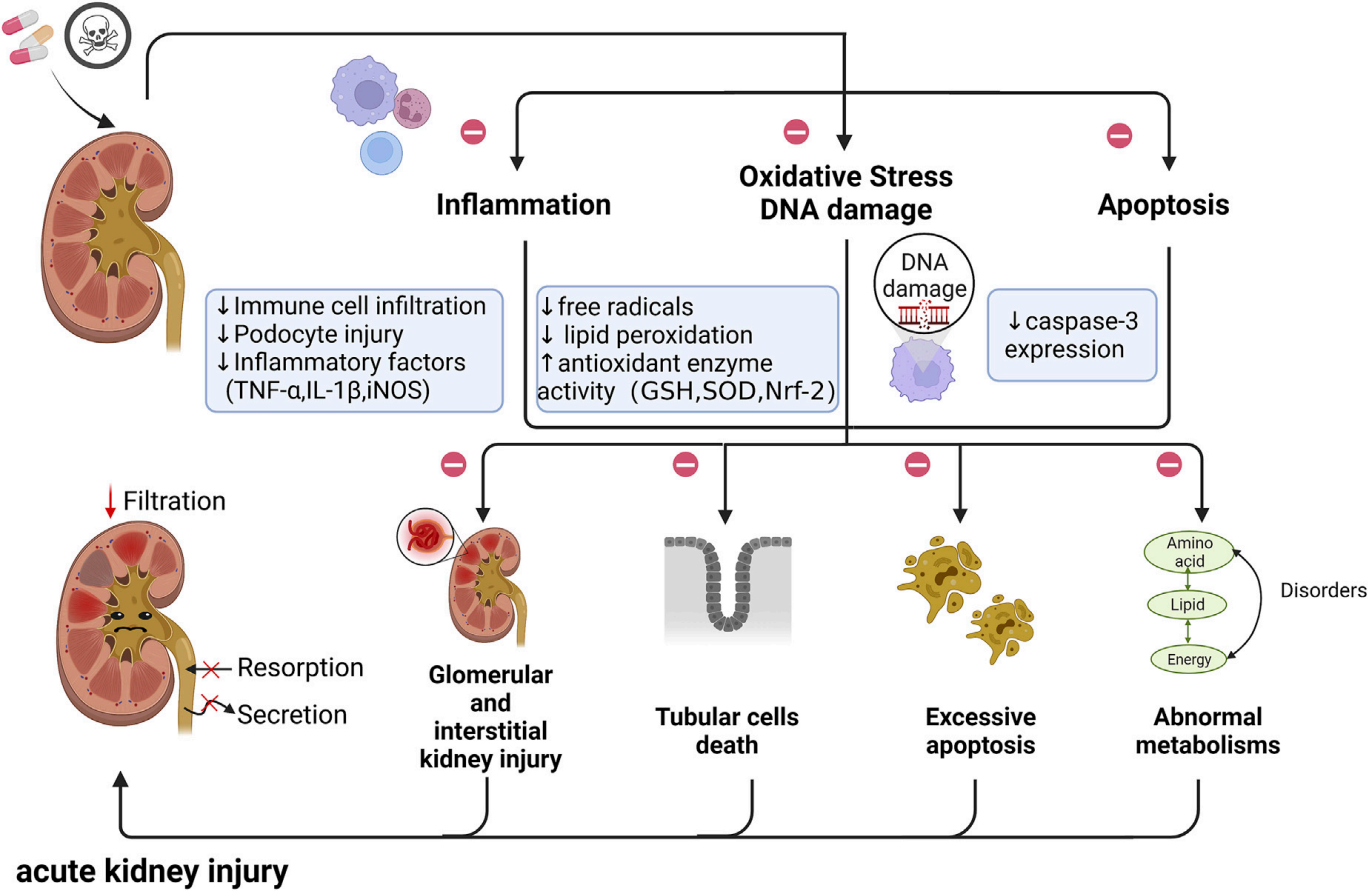
The potential role of renal toxins in the pathogenesis of AKI. Nephrotoxins may cause glomerular and interstitial kidney injury, tubular cell necrosis, and excessive apoptosis. In addition, the normal metabolism of the body can also be disrupted. This contributes to the reduction of kidney filtration and the impaired function of reabsorption and secretion, and eventually leads to acute kidney injury. (㊀ indicates therapeutic targets of quercetin; ↑ and ↓ indicates the regulatory role of quercetin on pathogenic changes); Abbreviations: TNF-α, tumor necrosis factor α; IL-1β, interleukin 1β; iNOS, inducible nitric oxide synthase; GSH, glutathione; SOD, superoxide dismutase; Nrf-2,Nuclear factor erythroid 2-related factor 2.

Specifically, with the development of industrial technology and the progress of science and technology, the damage of chemical raw materials to the kidney has grown immensely prominent. Data from animal models have shown that the protective effect of quercetin is closely related to the clearance of free radicals and reduction of lipid peroxidation in both industrial chemical raw materials and heavy metal and diesel particulate pollution (Li et al., 2016b; Qi et al., 2017; Uthra et al., 2017; Alshanwani et al., 2020; Morsi et al., 2022). Moreover, Quercetin was also found to reduce organophosphorus pesticide mixture-induced nephrotoxicity by regulating fatty acid, energy and sex hormone metabolism, protecting antioxidant defense systems and reducing DNA damage (Qi et al., 2017). Quercetin may regulate the metabolism of phospholipids, energy, fatty acids and amino acids to protect the kidney against acrylamide-induced nephrotoxicity (Bao et al., 2017; Bo et al., 2018). All these findings have shown that quercetin can produce significant protective effects in alleviating nephrotoxicity and renal insults caused by drug treatments (Figure 2).

### 4.2 Acute and chronic renal injury

#### 4.2.1 Acute kidney injury

Injuries from mesangial cells, endothelial cells (ECs), podocytes, TECs, and inflammatory cells could also lead to glomerular and interstitial fibrosis. Unresolved renal inflammation could also trigger cell apoptosis and fibrosis by releasing pro-apoptotic, pro-fibrotic growth factors, cytokines, and chemokines (Gu et al., 2021a).

Cell apoptosis and glomerular injuries are observed during renal ischemia. Quercetin can effectively prevent glomerular loss caused by renal hypochlorous ischemia (Gonçalves et al., 2021). The pathogenesis of renal ischemia/reperfusion injury (IRI) involves oxidative stress responses in the kidneys and distal organs, and the antioxidant effect of quercetin can prevent partial IRI (Gholampour and Sadidi, 2018). Regarding apoptosis, iron apoptosis is the iron-dependent regulatory necrosis that contributes to the progression of acute kidney injury (AKI), quercetin inhibits iron apoptosis in proximal renal TECs, thereby reducing AKI (Wang et al., 2021).

Carvedilol can relieve AKI caused by renal IRI and quercetin restores renal function by reducing inflammation (Rezk et al., 2021). Quercetin may also prevent AKI by regulating Mincle/Syk/NF-κB signaling to inhibit macrophage inflammation (Tan et al., 2020). Quercetin improves kidney damage by regulating macrophage polarization (Lu et al., 2018). Lipopolysaccharide (LPS) induces AKI in mice, and quercetin pretreatment protects mice from LPS-induced renal inflammation by inhibiting the TLR4/NF-κB signaling pathway (Tan et al., 2019). Quercetin may prevent sepsis-associated AKI by inhibiting NF-κB activation and upregulating Sirt1 expression (Lu et al., 2021). Besides, CD38 plays an important role in macrophage activation during sepsis-induced AKI. In the LPS-induced AKI mouse model, quercetin induces the blockade of CD38, thus significantly alleviating renal dysfunction and the infiltration of inflammatory cells (Shu et al., 2018).

Interestingly, as kidneys are one of the targets of SARS-CoV-2, up to 36% of SARS-CoV-2-infected patients develop AKI. COVID-19-induced inflammation is closely associated with AKI. Quercetin restores renal function by inhibiting the inflammatory and apoptosis-related signaling pathways (Gu et al., 2021b). Quercetin may potentially target SARS-CoV-2 3Clpro, which might inhibit the invasion of coronavirus, the life-threatening inflammation and cytokines storm in AKI (Diniz et al., 2020).

#### 4.2.2 Chronic kidney injury and renal fibrosis

One of the notable pathological characteristics of CKD is renal fibrosis, a prolonged wound-healing process that responds to multiple tissue injuries in the kidney. This process is characterized by glomerulosclerosis, tubular atrophy, and interstitial fibrosis. Studies have shown that renal fibrosis could be triggered by chronic inflammation. Renal injuries promote the recruitment of inflammatory cells and the release of related cytokines, chemokines, and ROS. This inflammatory process eventually activates fibroblasts and promotes the synthesis and accumulation of extracellular matrix (ECM) proteins. It is demonstrated that quercetin alleviated inflammation by upregulating the miR-124/NF-κB pathway in LPS-stimulated TECs (Guo et al., 2020). Of note, quercetin can also reduce macrophage accumulation and the expression of inflammatory cytokines in the kidneys of obstructive, therefore inhibiting renal fibrosis (Ren et al., 2016).

Transforming growth factor beta (TGF-β) is a major cytokine that promotes ECM accumulation. It may also induce the apoptosis of podocytes and promote epithelial to mesenchymal transition (EMT) progression (Arauz et al., 2015). One study has demonstrated that quercetin downregulated TGF-β signaling and reduced the expression of EMT-related proteins to halt the progression of glomerulosclerosis (Liu et al., 2019b). Other studies have also suggested that quercetin suppressed TGF-β signaling *via* Sonic Hedgehog, PTEN/TIMP3 and PI3k/Akt signaling pathways (Cao et al., 2018; Liu et al., 2019a; Tu et al., 2021). All the studies have suggested the anti-fibrotic role of quercetin in chronic kidney injury.

### 4.3 Diabetic nephropathy

#### 4.3.1 Anti-hyperglycemic effect

**FIGURE 3:**
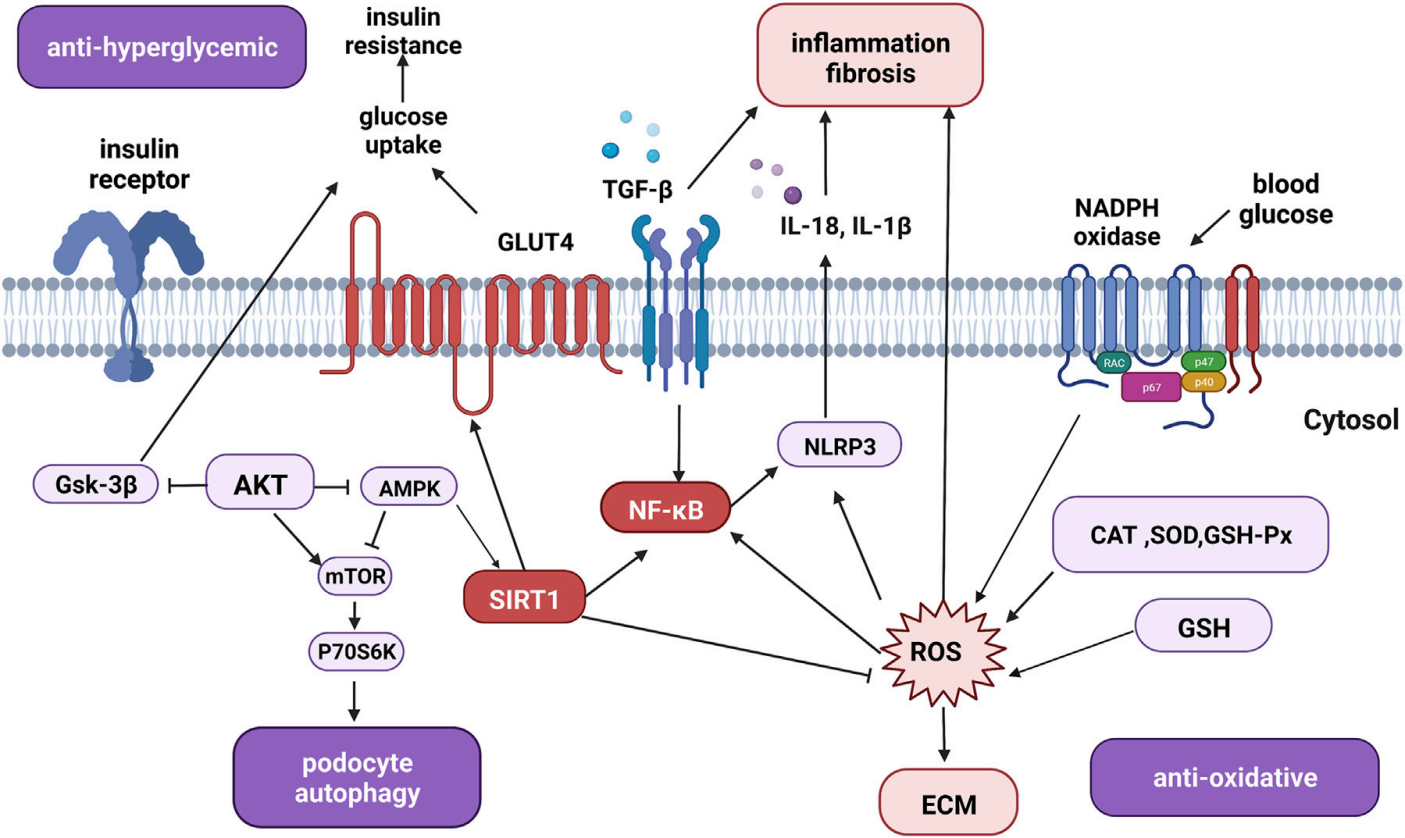
The overview of signaling pathways and therapeutic targets of quercetin in the treatment of diabetic nephropathy. Quercetin acts as an anti-hyperglycemic agent by regulating glucose-related signaling pathways. Quercetin also targets fibrotic, inflammatory, and oxidative mediators such as TGF-β, SIRT1, AKT, and NF-κB to inhibit inflammation, fibrosis, oxidative stress, apoptosis, and promote autophagy to exert renal protective effects. Abbreviations: Gsk-3β, glycogen synthase kinase-3; AKT, protein kinase B; AMPK, AMP-activated protein kinase; mTOR, mammalian target of rapamycin; P70S6K, 70-kDa ribosomal protein S6 kinase; GLUT4, glucose transporter protein type-4; TGF-β, transforming growth factor beta; NF-κB, nuclear factor κ-light-chain-enhancer of activated B cells; SIRT1, silent information regulator 1; NLRP3, NLR family pyrin domain containing 3; CAT, catalase; SOD, superoxide dismutase; GSH-Px, glutathione peroxidase; GSH, glutathione; ROS, reactive oxygen species; ECM, extracellular matrix; IL-18, interleukin-18; IL-1β, interleukin-1β; (Figure created with BioRender.com).

As shown in Figure 3, the regulatory roles of the signaling pathways involved in diabetic nephropathy (DN) are complex. Both hyperglycemia and dyslipidemia can induce structural and functional damage in diabetic kidneys (Sun et al., 2019). On one hand, quercetin decreases blood glucose levels by increasing the release of insulin while reducing hepatic glucose production. Mechanistically, quercetin may also enhance glucose uptake by regulating the expression and function of GLUT4 and the insulin receptor beta subunit (Ali et al., 2020).

On the other, hyperglycemia also induces metabolic alterations, resulting in the disturbance of protein, fat, and carbohydrate metabolism. Disorders of these metabolites also increase the burden on the diabetic kidneys. Quercetin at a dosage of 10 mg/kg/d can reduce blood glucose and triglycerides serum levels (Gomes et al., 2015). Likewise, at the early stage of DN, 50 or 100 mg/kg/d quercetin could improve lipid metabolism by alleviating albuminuria and renal function. In terms of lipid metabolism, quercetin reduces serum cholesterol, and triglycerides, and increases low-density lipoprotein cholesterol through the SCAP-SREBP2-LDLr signaling pathway in the diabetic rat model (Jiang et al., 2019).

#### 4.3.2 Anti-oxidative effect

The excessive expression of intracellular ROS is one of the significant changes in DN. ROS induces the activity of apoptosis-related enzymes, causing damage to the podocytes and promoting the proliferation of fibrotic cells to induce the synthesis of ECM (Ma et al., 2018). These processes result in renal fibrosis and inflammation and turn out to the progression of DN. Quercetin has acted as a free radical scavenger in DN animal models. For instance, one study measured the antioxidant-related enzymes and histopathological changes in kidneys and found that quercetin alleviated the damage by preventing oxidative stress (Elbe et al., 2015). As reported by other studies, dihydro quercetin exerts a renal protective effect on DN rats at the dose of 100 mg/kg/day, with the downregulated expression of ROS-related proteins and NLRP3 inflammasome (Ding et al., 2018). Besides, another study has revealed that both quercetin and quercetin-nanoparticle complex reduced structural damage to the kidney, improved renal function and alleviated oxidative stress by downregulating the expression of ICAM-1 (Tong et al., 2017).

#### 4.3.3 Autophagy promotion

Autophagy plays a crucial role in the intracellular degradation system for cellular homeostasis. As for kidney diseases, autophagy may protect functions in both glomerular and tubular compartments by suppressing excessive inflammation and fibrosis in AKI, CKD, and DN (Kimura et al., 2017; Bhatia and Choi, 2020). For example, hyperglycemia induces the dysregulation of autophagy in major types of resident kidney cells, mainly the impairment of podocytes. Autophagy is primarily regulated by signaling pathways such as the serine/threonine protein kinase mammalian target of rapamycin (mTOR), AMP activated protein kinase (AMPK), and sirtuins. High glucose can inhibit podocyte autophagy through AMPK pathway (Platé et al., 2020) and activate the mTOR signaling to inhibit podocyte autophagy. Evidence has suggested a quercetin-rich fruit, guava, is able to protect against type 2 diabetes mellitus-induced renal and pancreatic dysfunction by preventing cell apoptosis, autophagy, and pyroptosis (Lin et al., 2016). More studies have also demonstrated quercetin regulating blood glucose/lipid levels and improving renal fibrosis, potential mechanisms could be the modulation of the AMPK-dependent autophagy process, inhibition of mTORC1/p70S6K signaling, or the activation of Hippo pathways *in vitro* and *in vivo* (Lu et al., 2015; Lei et al., 2019; Lai et al., 2021). Further studies should focus on the glycemic regulating role and underlying mechanisms of quercetin treatment on DN.

### 4.4 Senolytic therapy for kidney disease

As clinical interest in kidney aging rapidly arises, the progression of cellular senescence relates closely to the stable cell cycle arrest. The accumulation of renal senescent cells (SCs) promotes inflammation and fibrosis, leading to multiple kidney disorders. The senolytics are a class of drugs that may selectively clear SCs. Quercetin, together with dasatinib, acts as the novel pharmacological senolytic agent for a number of kidney diseases (Kirkland and Tchkonia, 2020).

Senescent TECs are the driving force in renal fibrosis progression, which may activate fibroblasts. The combination of quercetin and dasatinib may specifically induce apoptosis of senescent TECs, therefore restoring renal function and ameliorating fibrosis (Li et al., 2021a). Another study has also shown that the combination of quercetin and dasatinib can alleviate renal insufficiency and damage in animal models of renal ischemia. *In vivo* study has revealed that senolytic therapy of quercetin and dasatinib improved renal artery stenosis by reducing the p21 positive stenotic TECs and attenuating mesenchymal transition (Kim et al., 2021). Notably, obesity could promote cellular senescence and impair renal function. Researchers have found an increased expression of renal markers of senescence, such as p16, p19, and p53, in a high-fat-diet-induced mouse model. Renal function and fibrosis are improved in quercetin-treated mice (Kim et al., 2019). Similarly, an open-label Phase 1 pilot study (NCT02848131) in patients with diabetic kidney disease showed that the combination of quercetin and dasatinib can eliminate senescent cells and significantly reduce senescent cell burden in adipose and skin tissues within 11 days. The possible mechanisms of their protective effects may be associated with the decrease of p16-, p21 expressing cells and the downregulation of senescence-associated secretory phenotype (including the expression of pro-inflammatory cytokines IL-6, IL-1α, and MMP-9) (Hickson et al., 2019). More explorations are needed to investigate the mechanism of analytic therapy and verify its efficacy and safety. The combination of quercetin and dasatinib could serve as new therapeutic agents to hinder renal senescence.

### 4.5 Other renal disorders

Dietary intake of the flavonoid quercetin has been proven effective in lowering blood pressure and restoring endothelial dysfunction in animal models of hypertension. Quercetin intake improves endothelium-dependent relaxation and inhibits α1-adrenoceptor mediated contractions in aortic rings from hypertensive rats. In addition, quercetin treatment in high dose promotes a significant reduction in blood pressure in spontaneously hypertensive rats compared to the control group (Choi et al., 2016; Elbarbry et al., 2020). These data not only demonstrate the anti-hypertensive effect of quercetin but also provide evidence for its role as a novel cardioprotective compound.

Renal cell carcinoma (RCC) has become a common subtype of kidney cancer, which has the highest propensity to manifest as metastatic disease. We lack knowledge of the correlation between migration and invasion in RCC, thus few therapeutic options are available (Meng et al., 2015). Intriguingly, recent studies have found that quercetin has anti-tumor effects against diverse types of cancers *via* multiple signaling pathways (Zhu et al., 2018). For example, a study explored the anti-tumoral effect of a potential chemopreventive effect of quercetin, the combination of quercetin and anti-sense oligo gene therapy provides stronger suppressive effects on RCC cells rather than a solo treatment. These studies have provided the possibility of quercetin as a novel treatment for renal cancer (Meng et al., 2015).

Autosomal dominant polycystic kidney disease (ADPKD) is a monogenic disease characterized by the massive enlargement of fluid-filled cysts in the kidney. One study has found that quercetin dramatically inhibited the formation and growth of the cyst, suggesting that quercetin could hinder renal cyst progression and should be represented as a novel candidate strategy for the treatment of ADPKD (Zhu et al., 2018). Nevertheless, speaking of kidney stones, quercetin also reduces the reabsorption of sodium, calcium, and water, thereby preventing the formation of a kidney stone in the urinary tract (Nirumand et al., 2018).

## 5 Conclusion and future perspectives

Quercetin, an active compound from natural products, has shown a significant protective effect in various models of kidney diseases. However, most of the studies have reported observational results and phenotype changes rather than the mechanisms of action related to the crucial pathogenesis. Besides, although experimental research has focused on the therapeutic effects and mechanisms of quercetin, it could hardly be used in the clinical setting due to its poor solubility and low oral bioavailability. Nevertheless, further research on nanoparticles, liposomes, micelles, or novel materials is in urgent need to improve the drug delivery system of quercetin and bring this natural compound to the forefront of therapeutic agents for the treatment of kidney disease.
